# Distinct Kinetics in the Frequency of Peripheral CD4^+^ T Cells in Patients with Ulcerative Colitis Experiencing a Flare during Treatment with Mesalazine or with a Herbal Preparation of Myrrh, Chamomile, and Coffee Charcoal

**DOI:** 10.1371/journal.pone.0104257

**Published:** 2014-08-21

**Authors:** Jost Langhorst, Annika Frede, Markus Knott, Eva Pastille, Jan Buer, Gustav J. Dobos, Astrid M. Westendorf

**Affiliations:** 1 Department for Integrative Gastroenterology, Kliniken Essen-Mitte, University of Duisburg-Essen, Essen, Germany; 2 Department for Internal and Integrative Medicine, Kliniken Essen-Mitte, University of Duisburg-Essen, Essen, Germany; 3 Institute of Medical Microbiology, University Hospital Essen, University of Duisburg-Essen, Essen, Germany; Shanghai Jiao Tong University School of Medicine, China

## Abstract

**Background:**

We found the first evidence of the efficacy of a herbal treatment with myrrh, dry extract of chamomile flowers, and coffee charcoal for ulcerative colitis (UC). However, the impact of the herbal treatment on the CD4^+^ T-cell compartment, which is essential for both the induction of UC and the maintenance of tolerance in the gut, is not well understood.

**Aim:**

To analyze the frequency and functional phenotype of CD4^+^ T cells and of immune-suppressive CD4+CD25^high^ regulatory T cells (Tregs) in healthy control subjects, patients with UC in remission, and patients with clinical flare of UC.

**Methods:**

Patients in clinical remission were treated with either mesalazine or the herbal preparation for 12 months. The frequencies of whole CD4^+^ T cells, CD4^+^CD25^med^ effector T cells, and Tregs and the expression of Foxp3 within the CD4^+^CD25^hig^ Tregs were determined by flow cytometry at 6 time points. We determined the suppressive capability of Tregs from healthy control subjects and from patients in remission or clinical flare.

**Results:**

A total of 79 patients (42 women, 37 men; mean age, 48.5 years; 38 with clinical flare) and 5 healthy control subjects were included in the study. At baseline the frequencies of whole CD4^+^ T cells, CD4^+^CD25^med^ effector cells, and Tregs did not differ between the two treatment groups and the healthy control subjects. In addition, patients with UC in sustained clinical remission showed no alteration from baseline after 1, 3, 6, 9, or 12 months of either treatment. In contrast, CD4^+^ T cells, CD4^+^CD25^med^effector T cells, and Tregs demonstrated distinctly different patterns at time points *pre-flare* and *flare*. The mesalazine group showed a continuous but not statistically significant increase from baseline to *pre-flare* and *flare* (p = ns). In the herbal treatment group, however, the percentage of the CD4^+^ T cells was lower at *pre-flare* than at baseline. This decrease was completely reversed after *flare*, when a significant increase was seen (CD4^+^CD25^med^ *pre-flare*/*flare* p = 0.0461; CD4^+^CD25^high^ baseline/*flare* p = 0.0269 and *pre-flare*/*flare* p = 0.0032). In contrast, no changes in the expression of Foxp3 cells were detected within the subsets of CD4^+^CD25^high^ regulatory T cells. Of note, no alterations were detected in the suppressive capability of CD4^+^CD25^high^ regulatory T cells isolated from the peripheral blood of healthy donors, from patients in remission, or from patients with clinical flare.

**Conclusions:**

In patients with UC experiencing acute flare, the CD4^+^ T compartment demonstrates a distinctly different pattern during treatment with myrrh, chamomile extract, and coffee charcoal than during treatment with mesalazine. These findings suggest an active repopulation of regulatory T cells during active disease.

**Trial Registration:**

EU Clinical Trials Register 2007-007928-18/DE

## Introduction

Ulcerative colitis (UC) is a chronic relapsing inflammatory bowel disease. Although no definitive cure is available, the aims of treatment are induction of remission and prevention of relapse. As maintenance remission therapy, treatment with aminosalicylates such as mesalazine is well established; the treatment guidelines recommend it as the gold standard for UC for at least two years after induced remission [1]–[2].

Complementary and alternative medicine (CAM) is widely used for chronic diseases [3]–[8], and for UC herbal therapies are one of the most frequently used CAM treatment methods [5]–[9]. For more than 40 years a combination of myrrh, chamomile flowers, and coffee charcoal has been used in Germany as treatment for diarrhea. This treatment is well tolerated and exhibits a good safety profile [10]. Because of its composition, it is also promising both as a treatment for acute UC and as maintenance therapy. Myrrh resin, *Commiphora molmol*, with its main ingredients furanose sesquiterpene, diterpenoids, and volatile acids, exerts a broad spectrum of activity, including antiinflammatory, antiphlogistic, antioxidant, antibacterial, and astringent potentials [11]–[14]. Chamomile dry extract of chamomile flowers, with its main ingredients volatile acids, flavonglycosides, and hydroxycoumarins, exerts anti-inflammatory and antiphlogistic effects and has antibacterial, spasmolytic, and ulcer protective potentials [15]–[22]. In addition, coffee charcoal has antibacterial, astringent, and absorptive potentials [23].

We found first evidence of the potential efficacy of the herbal preparation of myrrh, chamomile, and coffee charcoal, which was found to be non-inferior to the gold standard of therapy, mesalazine, in a randomized controlled trial as maintenance therapy for UC patients [10]. However, not much is known about the mode of action of this therapy for patients with UC, especially in a clinical setting.

UC is believed to be, at least in part, a T cell–driven disease, and loss of immune tolerance in the gut plays an important role in its pathogenesis [24]. CD4^+^CD25^high^Foxp3^+^ regulatory T cells play a central role in the maintenance of tolerance in the gut and may also contribute to inadequate counterregulation in inflammatory bowel disease (IBD) [25]. This suggestion is supported by findings showing that patients with the immunodysregulation polyendocrinopathy enteropathy X-linked (IPEX) syndrome, characterized by a defect in the function of regulatory T cells, experience severe bowel inflammation as one component of the disease [26]. Moreover, studies of the peripheral blood of UC patients found a decrease in the relative and absolute numbers of CD4^+^ regulatory T cells [27]–[29]. However, the relative function of regulatory T cells during remission and flare is not entirely clear. It is likely that current therapeutic approaches influence the course of regulatory T cells and may even achieve their therapeutic success, at least in part, via interaction with regulatory T cells.

The present study was a sub-study of a randomized controlled trial comparing the efficacy of the herbal preparation of myrrh, chamomile extract, and coffee charcoal with that of the established therapy, mesalazine, in maintaining remission for 12 months in UC patients [10]. To gain information about the biological function of the drugs, we assessed the kinetics of whole CD4^+^ T cells, CD4^+^CD25^med^ effector T cells, and CD4^+^CD25^high^ regulatory T cells, as well as Foxp3 expression within CD4^+^CD25^high^ regulatory T cells, in the peripheral blood of UC patients during treatment with the herbal preparation or with mesalazine.

## Materials and Methods

The study was conducted from June 2008 to July 2010 as a single-center trial in the Department for Internal and Integrative Medicine, Kliniken Essen-Mitte, Germany. It was planned and performed as an independent sub-study of a drug trial comparing the efficacy of a herbal preparation of myrrh, dry extract of chamomile flowers, and coffee charcoal with that of mesalamine as treatment for maintaining remission in UC [10] (Trial registration: https://www.clinicaltrialsregister.eu/ctr-search/trial/2007-007928-18/DE; EudraCT number: 2007-007928-18). The study was conducted in accordance with the Helsinki Declaration and good clinical practice (GCP) guidelines. All patients gave written informed consent for participation, and the study protocol was approved by the Ethics Committee of the Medical Faculty, University of Duisburg-Essen.

### Inclusion and Exclusion Criteria

The criterion for inclusion was a diagnosis of ulcerative colitis, verified by the defining symptoms (rectal bleeding, diarrhea), colonoscopy, and histopathology. Patients eligible for the study had to be between 18 and 75 years of age and in self-reported clinical remission for 7 days to 12 months before beginning the study, as reported retrospectively at the time of the screening visit. In addition, patients had to be in clinical remission as defined by a score of less than 4 on the Colitis Activity Index (CAI) (Rachmilewitz) [30] at baseline.

Important exclusion criteria were clinically active disease, infectious or chronic active colitis, current use of antibiotics or corticosteroids, treatment with biologic agents or other immunosuppressive drugs (azathioprine, methotrexate), complete colectomy, relevant comorbidity, or pregnancy within the past 3 months.

Blood samples obtained from 5 healthy donors aged 31 to 56 years were used as controls.

### Study Medication

Patients were treated either with an oral preparation of 100 mg myrrh, 70 mg chamomile extract, and 50 mg coffee charcoal (Myrrhinil intest; Repha GmbH, Hannover, Germany) or with mesalazine, consisting of eudragit-L–coated 5-aminosalicylic acid (Salofalk 500 mg; Dr Falk Pharma GmbH, Freiburg, Germany). The test group received four tablets of the herbal preparation three times daily and one placebo tablet three times daily over the 12-month period. The control group received four placebo tablets three times daily and one tablet of Salofalk 500 mg three times daily over the 12-month period. No concomitant medication for UC was allowed throughout the study. In the event of a flare, treatment was initiated according to the guidelines for UC.

### Evaluation

#### Clinical disease activity

Clinical disease activity was determined by the CAI according to Rachmilewitz [30], which includes a combination of laboratory parameters and clinical symptoms: weekly calculation of bowel frequency, blood in stool, well-being, abdominal pain, fever, extraintestinal symptoms, erythrocyte sedimentation rate, and hemoglobin. A score of 4 or less indicates remission; a score higher than 4 indicates active disease.

The CAI was calculated at baseline; after 1, 3, 6, 9, and 12 months; and when the patient reported symptoms of an acute flare. In addition, a patient diary was maintained over the 12-month period. A modified CAI, including weekly calculation of bowel frequency, blood in stool, well-being, abdominal pain, fever, and extraintestinal symptoms, was used for monitoring the safety profile of the therapies.

#### Tests of blood and fecal samples

Blood samples and a fecal specimen were collected at baseline; after 1, 3, 6, 9, and 12 months; and whenever the patient reported symptoms of an acute flare. Blood samples were analyzed for white blood cell (WBC) count and serum c-reactive protein (CRP) concentrations; stool samples were analyzed for fecal calprotectin (ELISA, Immundiagnostik, Bensheim, Germany) as previously described [31].

#### Endoscopy

Sigmoidoscopy was performed at baseline and at the end of the 12-month period or in the event of a flare. An Endoscopy Index (EI) was calculated by an experienced endoscopist. The Endoscopic Index score (0–12) according to Rachmilewitz [30] included four categories: granulation scattering reflected light (0–2), vascular pattern (0–2), vulnerability of mucosa (0–4), and mucosal damage (0–4). A summated score of 1 or below indicated mucosal healing.

#### Definitions of time points

Baseline measures were determined at study entry. The time point *flare* was defined by a CAI score higher than 4 and was confirmed by sigmoidoscopy and by WBC count and levels of CRP and calprotectin. The time point *pre-flare* was defined as the last predefined time point in clinical remission before a flare was confirmed.

#### Isolation of peripheral blood mononuclear cells

The frequencies of various T-cell subsets in peripheral blood mononuclear cells (PBMCs) were determined at the various predefined time points and in the event of a flare. PBMCs were isolated from heparin-treated blood by Bicoll (Biochrom, Germany) density gradient centrifugation (Biochrom AG, Berlin, Germany). Isolated cells were washed with buffer and were either analyzed immediately by flow cytometry or cryopreserved in medium containing 10% fetal calf serum (FCS; PAA Laboratories GmbH, Pasching, Austria) and 10% dimethyl sulfoxide (DMSO; Carl Roth GmbH, Karlsruhe, Germany).

#### Antibodies and flow cytometry

PBMCs were stained with fluorochrome-labeled anti-CD4 and anti-CD25 antibodies (both from Miltenyi Biotec, Germany). Intracellular staining was performed with the Foxp3 staining kit from eBioscience (NatuTec, Frankfurt, Germany) according to the manufacturer‘s recommendations. In brief, after surface staining, cells were washed, suspended in Fix/Perm solution (eBioscience), and incubated at 4°C for 90 min. Samples were washed with a washing buffer and then washed twice more with a permeabilization buffer (eBiosciences). Cells were then stained with fluorochrome-labeled anti-Foxp3 antibody in a permeabilization buffer for 30 min at 4°C. After washing, flow cytometric analyses were performed with a FACSCalibur flow cytometer and CellQuest software (BD Biosciences, Heidelberg, Germany). Cryopreserved PBMCs from a healthy donor with well-defined expression of CD4, CD25, and Foxp3 were included as internal staining controls.

#### Suppression assay

CD4^+^CD25^high^ T cells and CD4^+^CD25^−^ T cells were sorted from PBMCs with a FACSAria II cell sorter (BD Biosciences). CD4^+^CD25^−^ responder T cells (3–4×10^4^, depending on the blood donor) were labeled with carboxyfluorescein diacetate succinimidyl ester (CFSE) (Invitrogen) and either cultured alone or co-cultured with CD4^+^CD25^high^ regulatory T cells (1.5–2×10^4^) from the same donor at a ratio of 2 to 1 in the presence of Treg Suppression Inspector (Miltenyi Biotec) for 3 days. All cells were cultured in Iscove's Modified Dulbecco's Media (IMDM) with GlutaMAX™ (Invitrogen) supplemented with 10% heat-inactivated FCS (PAA Laboratories, Pasching, Austria), 25 mM HEPES (Biochrom, Berlin, Germany), 100 U/mL penicillin, and 0.1 mg/mL streptomycin (both Sigma-Aldrich). Proliferation of gated CD4^+^CD25^−^ responder T cells was assessed by loss of the fluorescent CFSE.

#### Statistical analyses

Statistical analyses were performed with the Statistical Package for the Social Sciences (SPSS, Inc., Chicago, IL, U.S.A., version 20.0) software and Prism 5.0 software (GraphPad, La Jolla, CA). Descriptive statistics for this exploratory study were computed according to group of diagnosis, and groups were compared by one-way ANOVA, repeated-measure ANOVA followed by multiple comparison tests, or paired Student’s *t* tests. Statistical significance was set at the level of *P*<0.05.

## Results

### Patients

A total of 126 patients with UC in clinical remission were screened for the study. Of these, 30 did not meet the inclusion criteria or met one or more of the exclusion criteria. A total of 96 patients (51 women; 53.1%) took study medication for at least one week and were followed up for 12 months. The first 79 consecutive patients from this patient group were included in the presented sub-study (42 women; mean age, 48.5 years [range, 20 to 75 years]). Baseline characteristics are shown in Table 1. In addition, 5 healthy control subjects (2 women, 3 men; mean age, 43 years [range, 31 to 56 years]) were included in the study.

**Table 1:** Demographic data and pre-study clinical characteristics for the two treatment methods (mesalazine and herbal preparation) and the healthy control group.

	Mesalazine	HerbalPreparation	*P* value	Control
Characteristics	(n = 39)	(n = 40)		
Men/Women	19/20	18/22	ns	3/2
Mean Age (y)	45.2±13.0	51.0±14.0	0.062	43.0±12.0
Montreal Classification [57] *				
E1 Proctitis	7	5	ns	
E2 Left-sided colitis	22	20	ns	
E3 Extensive colitis	10	15	ns	
Mean Duration of ulcerative colitis (y)	11.3±9.5	12.7±10.7	ns	
CAI≤4	39	40		
CAI (mean)	0.9±1.0	0.8±1.0	ns	
Endoscopy Score (mean)	1.0±1.4	1.1±1.5	ns	
Endoscopy Score without	79%	73%	ns	
signs of acute inflammation				
Taking oral 5-ASA maintenance medication at baseline	31	22	0.046	

ns = not significant.

*E1 Ulcerative proctitis: Involvement limited to the rectum (that is, proximal extent of inflammation is distal to the rectosigmoid junction). E2 Left-sided UC (distal UC): Involvement limited to a portion of the colorectum distal to the splenic flexure. E3 Extensive UC (pancolitis): Involvement extends proximal to the splenic flexure.

### Clinical Course of Disease

Of the 79 patients, 39 were treated with mesalazine and 40 were treated with the herbal preparation. Thirty-eight patients experienced a clinical flare according to the predefined criterion of CAI score higher than 4: 18 (46.2%) of those treated with mesalazine and 20 (50%) of those treated with the herbal preparation.

### Blood and Fecal Tests

Blood from the 79 included patients was analyzed for WBC count and serum CRP concentration, and stool samples were analyzed for fecal calprotectin at the predefined time points. Comparing the two treatment groups treated with either mesalazine or the herbal preparation no difference (p = ns) could be shown at any time point regarding CRP, WBC or fecal calprotectin.

Comparing the group of all patients in an acute flare (time point flare) to the group of all patients in sustained remission (time point 12 month) mean CRP levels (remission: 0.4±0.4 mg/dl; acute flare: 1.4±2.2 mg/dl; *p* = 0.002), WBC (remission: 6.6±2.0/nl; acute flare: 8.0±2.9/nl; *p* = 0.005) and mean calprotectin levels (remission: 15.2±15.0 µg/g; acute flare: 37.6±70.0 µg/g; p = 0.007) were significantly elevated in the group of patients in an acute flare.

### Endoscopy

Of the 79 included patients in clinical remission who underwent sigmoidoscopy at baseline, 19 had an endoscopy sum score higher than 1 (11 patients in the herbal treatment group; 8 in the mesalazine group).

Of the 38 patients who experienced a flare, 32 underwent sigmoidoscopy. Sixteen of the 17 patients in the herbal treatment group and all 15 patients in the mesalazine group had an endoscopy sum score higher than 1, indicating active inflammation.

At the end of the 12-month period, 37 of the 41 patients in sustained clinical remission underwent a closing endoscopy. Thirteen of the 16 patients in the herbal treatment group (81.3%) and 19 of 21 patients in the mesalazine group (90.5%) had an endoscopy sum score of 1 or lower, indicating mucosal healing.

The Rachmilewitz Endoscopic Activity Index sum score was significantly higher (*P*<0.0001) in the group with an acute flare (6.4±2.0) than at baseline (1.1±1.4) and at 12 months (1.0±1.0).

### Peripheral Cd4^+^ T-Cell Subsets

Staining studies were completed on the blood of 75 of the 79 included patients and on the blood of the 5 healthy control subjects. Staining studies failed for 4 of the included patients.

The complete set of blood samples could be evaluated for 66 patients and could be included in the final analysis: 16 patients in the herbal treatment group and 11 patients in the mesalazine treatment group who experienced a flare during the 12-month period; and 19 patients in the herbal treatment group and 20 patients in the mesalazine treatment group who showed sustained clinical remission during the 12-month period.

### Baseline

**Figure 1:**
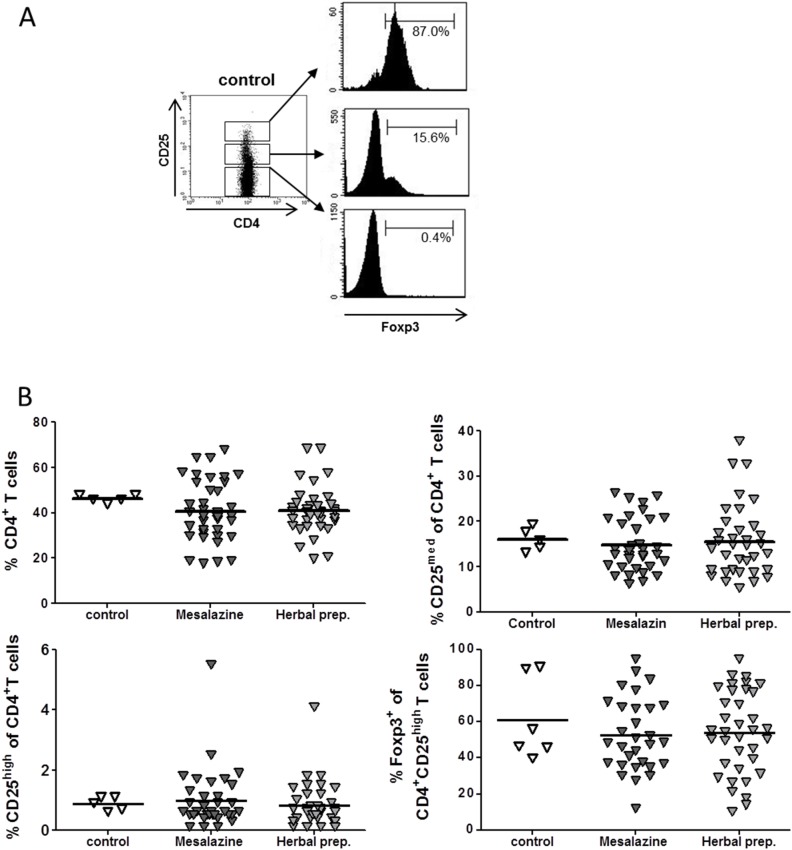
Baseline frequency of CD4^+^ T-cell subsets in the peripheral blood of patients with ulcerative colitis in remission and healthy donors as determined by flow cytometry for the expression of CD4 and CD25 and intracellular Foxp3. (A) Gating strategy for CD4 and CD25 expression is shown as a dot plot. Expression of Foxp3 by indicated CD4^+^ T cell subsets demonstrated as histograms. (B) Baseline percentage of indicated T-cell subsets among CD4^+^ T cells in healthy controls and patients with ulcerative colitis in remission before treatment with mesalazine or herbal preparation. Means are shown as scatter plot. Mean values are indicated by lines.

To define the baseline characteristics of CD4^+^ T cell subsets in this study, we determined the percentage of CD4^+^ T cells, CD4^+^CD25^med^ effector T cells, and CD4^+^CD25^high^ regulatory T cells and the expression of Foxp3 within the CD4^+^CD25^high^ subsets in the peripheral blood of patients with UC in remission. Interestingly, the results demonstrated no differences at baseline between the two treatment groups and the healthy control subjects (Fig. 1).

### Patients in Sustained Clinical Remission

**Figure 2:**
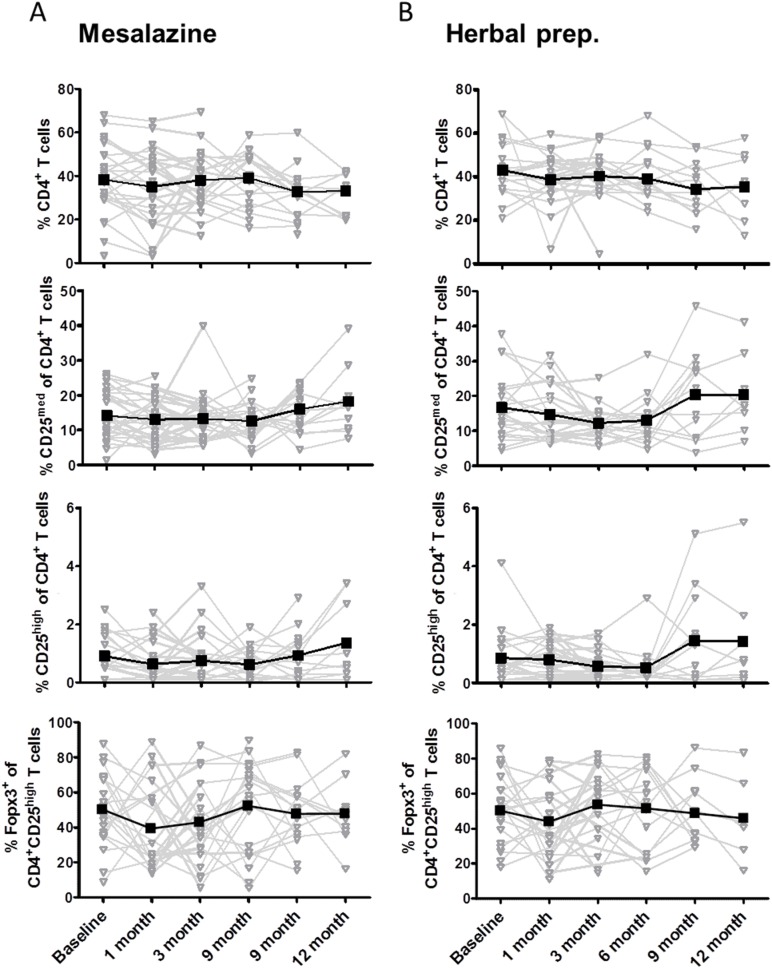
Frequency of CD4^+^ T-cell subsets in the peripheral blood of patients with ulcerative colitis in *sustained clinical remission*. PBMCs were isolated from heparinized blood samples from patients treated with mesalazine or a herbal preparation at baseline and 1, 3, 6, 9, and 12 month after baseline. The expression of CD4, CD25, and intracellular Foxp3 as determined by flow cytometry is shown as the percentage of indicated T-cell subsets under mesalazine treatment (A) or herbal preparation treatment (B) per patient (gray). Mean value per time point is shown in black.

In the patient group with sustained clinical remission, there was no significant difference between the baseline observation and the 12-month observation for both treatment groups in terms of peripheral CD4^+^ T cells, CD4^+^CD25^med^ effector T cells, CD4^+^CD25^high^ regulatory T cells, and Foxp3 expression (Fig. 2).

### Patients Experiencing a Clinical Flare

**Figure 3:**
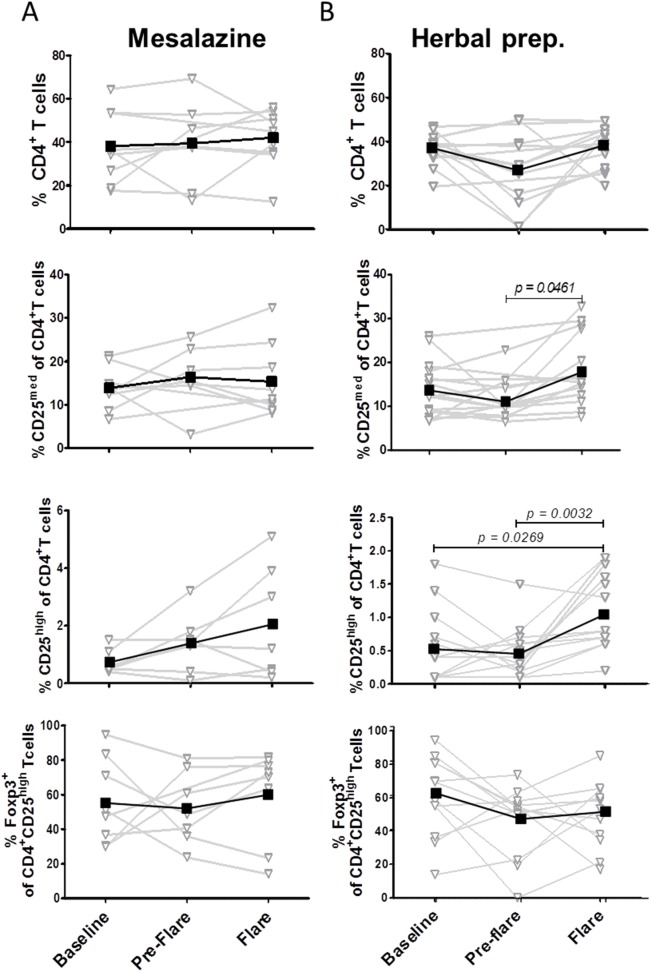
Frequency of CD4^+^ T-cell subsets in the peripheral blood of patients with ulcerative colitis experiencing *a clinical flare*. PBMCs were isolated from heparinized blood samples from patients with ulcerative colitis treated with mesalazine (A) or herbal preparation (B) at indicated time points. The expression of CD4, CD25, and intracellular Foxp3 was determined by flow cytometry. Results are demonstrated as kinetic per patient (gray). Mean value per time point is shown in black.

For the group of patients experiencing a clinical flare, we focused on the last observation time point before flare (*pre-flare*) and clinical *flare*. PBMCs were isolated from heparinized blood samples taken from UC patients treated with mesalazine (n = 9) or with the herbal preparation (n = 14) at the indicated time points. Interestingly, in the summary of all patients we detected an increase in the frequency of the various CD4+ T-cell subsets. However, the significant changes were based on the alteration in the group of patients treated with the herbal preparation. The mesalazine group showed a continuous but not significant increase in the percentage of CD4^+^CD25^med^ effector T cells and CD4^+^CD25^high^ regulatory T cells from baseline to *pre-flare* to *flare* (*P = *not significant) (Fig. 3A). In the herbal treatment group a significant change could be shown for CD4^+^ T cells and for the subgroups of CD4^+^CD25^med^ effector T cells and CD4^+^CD25^high^ regulatory T cells. The percentage of CD4^+^ T cells was lower during *pre-flare* than at baseline (Fig. 3B). This reduction was completely abolished and reversed at time point *flare*. In addition, in the herbal treatment group the percentage of CD4^+^CD25^med^ effector T cells was significantly higher at time point *flare* than at the time point *pre-flare*, and the percentage of CD4^+^CD25^high^ regulatory T cells was significantly higher than at baseline or during *pre-flare* (CD4^+^CD25^med^ *pre-flare vs flare, P = 0.0461*; CD4^+^CD25^high^ baseline *vs flare*, *P = 0.0269*; *pre-flare vs flare, P = 0.0032*). However, the expression of Foxp3 within the CD4^+^CD25^high^ subsets was not significant altered during baseline, *pre-flare*, and *flare* (Fig. 3) in the mesalazine and herbal treatment groups.

### Regulatory Function of CD4^+^CD25^high^ T Cells during Remission and Clinical Flare

**Figure 4:**
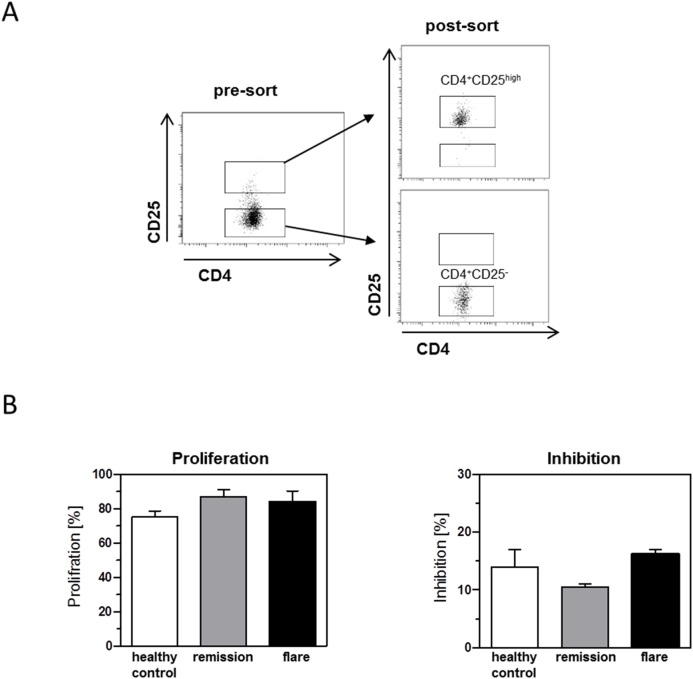
Regulatory function of CD4^+^CD25^high^ T cells during remission and clinical flare. PBMCs from healthy donors and from patients with ulcerative colitis were stained for CD4 and CD25 expression. CD4^+^CD25^−^ and CD4^+^CD25^hgh^ regulatory T cells were sorted by flow cytometry. (A) Gating strategy and purity of sorted CD4^+^CD25^−^ and CD4^+^CD25^high^ cells are demonstrated as dot plot. (B) CD4^+^CD25^−^ responder T cells were co-cultured at a ratio of 2 to 1 with or without autologous CD4^+^CD25^hgh^ regulatory T cells under stimulation with Treg suppression inspector beads. The proliferation of responder cells was measured by the loss of CFSE dye, and inhibition was calculated.

To determine whether the CD4^+^CD25^high^ regulatory T cells isolated from patients in remission and from patients with clinical flare are functional, we performed suppression assays. CD4^+^ T cell subsets were sorted as described in Figure 4A. CD4^+^CD25^high^ T cells were co-cultured with autologous CD4^+^CD25^−^ responder T cells stimulated for 3 days. Proliferation of gated CD4^+^CD25^−^ responder T cells was assessed by flow cytometry. Interestingly, CD4^+^CD25^high^ T cells isolated from healthy control subjects, patients in remission, and patients with clinical flare showed similar suppressive activity, demonstrating the functional activity of regulatory T cells in the peripheral blood of patients with clinical flare (Fig. 4B).

## Discussion

This paper presents three messages that we believe are important. First, the results introduce the course of CD4^+^CD25^high^ regulatory T cells in the peripheral blood of patients with UC in remission or with active disease over a period of 12 months. Second, relevant changes in the course of peripheral CD4^+^CD25^high^ regulatory T cells in peripheral blood could be shown in the patient group that experienced a clinical flare, but no differences in the suppressive activity of these regulatory T cells were detected between remission and active disease. Finally, these changes demonstrated a distinctly different pattern between the herbal treatment group and the mesalazine group, and these different patterns may allow the identification of a different mode of action for the herbal treatment.

Various studies have shown that herbal medicine is a relevant CAM treatment method for IBD. Herbal medicine is the most commonly used form of CAM for IBD and is used by as many as 58% of patients [5]; [7]; [32]–[33]. However, many of the potentially used herbs have not been scientifically studied as treatment for IBD, and little is known about their mode of action in UC, especially in a clinical setting.

In contrast, since their development in 1940 aminosalicylates have had a long and storied history in the treatment of UC. As shown in placebo-controlled trials, maintenance therapy is essential for UC because the relapse rate is 5 times higher for untreated UC than for treated disease [34]–[36]. Mesalazine-based agents exert various effects on leukotrienes, cytokines, and oxygen radicals [37]–[38]. Whittle and coworkers [38] described the complex biochemical and pharmacological profile of these agents, including their ability to inhibit intestinal macrophage chemotaxis [39] and to reduce the secretion of antibodies from mononuclear cells [40]. Furthermore, 5-ASA can reduce the release of proinflammatory cytokines [41], and aminosalicylates can inhibit the arachidonate lipoxygenase and cyclooxygenase pathways [42]. In addition, there is first evidence for the potential of aminosalicylates as antioxidants and free radical scavengers [43]–[44]. However, the definitive mechanisms underlying the anti-colitic properties of the aminosalicylates have still not been fully identified.

Treatment with the herbal preparation of myrrh, chamomile extract, and coffee charcoal offers a potential multitarget approach. The herbal preparation combines the anti-inflammatory, antiphlogistic, and antibacterial potential of Myrrh resin, *Commiphora molmol* [11]–[14], and chamomile dry extract of chamomile flowers [15]–[22] with the antibacterial, astringent, and absorptive potential of coffee charcoal [23].

Published studies have discussed the various anti-inflammatory effects of the herbal ingredients. For example, Cheng and co-workers demonstrated that a myrrh methanol extract exerts an inhibitory effect on the production of NO, PGE2, IL-1β, and TNF-α by down-regulating inducible nitric oxide synthase and COX-2 gene expression via haem oxygenase–1 protein synthesis induction in LPS-stimulated macrophages [45]. Bhaskaran and co-workers showed that murine macrophages pretreated with chamomile are protected from cell death caused by H_2_O_2_. Chamomile exposure significantly increases the expression of antioxidant enzymes haem oxygenase-1, peroxiredoxin-1, and thioredoxin-1 in a dose-dependent manner [46].

The results of our randomized, double-blind, double-dummy study over a 12-month period in UC patients showed that the herbal preparation of myrrh, chamomile extract, and coffee charcoal is well tolerated and exhibits a good safety profile [10]. In addition, we found first evidence that the efficacy of this treatment is non-inferior to that of the gold standard therapy mesalazine as maintenance therapy for UC patients [10]. However, the therapeutic effect of the two treatment methods may be introduced via different modes of action.

CD4^+^CD25^high^ regulatory T cells play a central role in maintaining tolerance in the gut. They are crucial in maintaining immune homeostasis and establishing tolerance to foreign, non-pathogenic antigens, including those found in commensal bacteria and food [47]. Because of their potent, antigen-specific suppressive capability, CD4^+^CD25^high^ regulatory T cells have special relevance for the course of disease in IBD and may be promising candidates for immune therapy in a variety of chronic inflammatory diseases, including IBD [47]. In fact, in multiple animal models regulatory T cells have been shown to be effective in both the cure and the prevention of experimental colitis [48]–[51]. For example, the transfer of regulatory T cells into mice with colitis leads to resolution of the lamina propria infiltrates and reappearance of normal intestinal architecture [52]. Although further investigation is warranted, it has been proposed that active IBD is particularly associated with a quantitative shift rather than a functional defect of regulatory T cells [24]. Therefore, it has been proposed that the relative or absolute number of regulatory T cells plays a crucial role in the prevention and therapy of UC.

Current treatment strategies for IBD rely on the use of non-specific immunosuppressive or anti-inflammatory agents. Because evidence to date suggests that regulatory T cells are indeed functional in IBD patients, expansion of autologous cells might be a feasible therapeutic approach in the future. Well in line with this, we showed that regulatory T cells from healthy donors and UC patients exert similar suppressive activity. Antigen-specific regulatory T cells may offer an effective therapy through specific and potent targeting of the response to disease-driving antigens at the site of inflammation [47]. It is, however, likely that current immunosuppressive or anti-inflammatory approaches influence the course of regulatory T cells, and it may even be possible that they achieve their therapeutic success at least in part via interaction with regulatory T cells. Maul *et al.* reported a decrease in the frequency of regulatory T cells in the peripheral blood and an increased frequency in mucosal lymphoid tissues in IBD patients with active disease [53]. Regrettably, no information about current medication use was given in this study. However, a recent study involving pediatric patients with IBD demonstrated a significant increase in circulating CD4^+^ regulatory T cells independent of disease activity. In this study, 24 UC patients were included: 5 did not receive therapy, 5 were treated with immunosuppressive drugs, 16 were treated with anti-inflammatory drugs, and 1 received anti-TNF therapy [54].

Only recently, first findings were published about the impact of anti-inflammatory or immunosuppressive drugs on CD4^+^CD25^high^ regulatory T cells in patients with IBD. Veltkamp and colleagues reported a decrease in the frequency of regulatory T cells in the peripheral blood and an increased frequency in mucosal lymphoid tissues in IBD patients with active disease; this decrease was partially reversed with anti-TNFα treatment and a decrease in disease activity [55]. In the study by Li and co-workers, infliximab therapy resulted in a significant and sustained relative increase in the numbers of CD4^+^CD25^high^ regulatory T cells in peripheral blood, particularly in responders [56].

Our study investigated the course of CD4^+^ T cells, CD4^+^CD25^med^ effector T cells, and CD4^+^CD25^high^ regulatory T cells under maintenance therapy with mesalazine or herbal therapy with myrrh, chamomile extract, and coffee charcoal. The numbers of CD4^+^CD25^high^ regulatory T cells showed a slight but not statistically significant increase during flare in the mesalazine group. However, most remarkably, a significant change could be shown for CD4^+^ T cells, CD4^+^CD25^med^ effector T cells, and CD4^+^CD25^high^ regulatory T cells in the herbal treatment group. The special study setting introducing the time point *pre-flare* was crucial for elucidating the special course of the T-cell populations. The percentage of CD4^+^ T cells was lower during *pre-flare* than at baseline. A possible explanation for this effect might be an active pooling to the intestine, indicating activation of disease before the clinical onset. This decrease was completely abolished and reversed at time point *flare*; compared with the time point *pre-flare*, at *flare* a significant increase in the number of CD4^+^CD25^med^ effector T cells and an even more marked increase in the number of CD4^+^CD25^high^ regulatory T cells was found in the herbal treatment group. This finding might be interpreted as an active repopulation of newly generated regulatory T cells during active disease.

Currently, it remains speculative whether the pattern of the T-cell population in the herbal treatment group is a more natural course of regulatory T cells. It is likely, but not yet confirmed, that herbal therapy with myrrh, chamomile extract, and coffee charcoal achieves treatment success not via relevant plasma levels but via direct effects at the mucosa. In contrast, published studies describe a broader spectrum including more systemic effects for mesalazine, and these findings may counteract a more distinct pattern of regulatory T cells in peripheral blood in the event of a flare. Hence, although the cellular or molecular mechanisms are not yet fully understood, these findings suggest at least in part a presumably different mechanism of action between the herbal therapy and mesalazine. These findings might merit investigation of the combination of the two treatment options for patients with ulcerative colitis. In addition, the significant decrease in the number of CD4^+^ T cells before the clinical onset of flare, as well as the significant increase in the number of CD4^+^CD25^med^ effector T cells and the even more marked increase in CD4^+^CD25^high^ regulatory T cells during active disease when compared to the time point *pre-flare,* should be subjected to further investigation of the pathophysiological concepts in UC.

Our study had several important limitations. First, it was designed primarily as an efficacy trial, and the presented findings are the results of an exploratory analysis. Second, flare was defined clinically by the use of a clinical index, which involved patients’ subjective interpretation of symptoms rather than objective signs of inflammation. However, in most instances flare was confirmed by endoscopy and by elevated levels of blood and fecal inflammation markers, thus providing a more objective definition of acute inflammation. Finally, because the herbal preparation shows a slight trend toward lower efficacy [10], one could argue that the specific pattern may reflect a flare rather than a primary mechanism. However, the differences in T-cell populations during flares indicate a primary failure of the applied therapy, which is true for both investigated treatments.

In conclusion, in UC patients experiencing an acute flare, populations of T cells and especially CD4^+^CD25^high^ regulatory T cells demonstrate a distinctly different pattern in response to treatment with the herbal preparation of myrrh, chamomile extract, and coffee charcoal than they demonstrate in response to treatment with mesalazine. These findings suggest an active repopulation of regulatory T cells during active disease. However, further studies are needed to clarify the mode of action of herbal treatment.
